# A Report of Five Cases of Colles Fracture, Treated after the Plan of Prof. E. M. Moore

**Published:** 1873-09

**Authors:** B. L. Hovey

**Affiliations:** Rochester, N. Y.


ART. II.—*A report of Five Cases of Colles Fracture, treated after the plan of* Prof. E. M. Moore, M. D. By Dr. B. L. Hovey, of Rochester, N. Y.

Mrs. V., aged 24 years, was thrown from a Carriage, June 19th, 1872, and striking on the palm of the right hand, fractured the radius one inch from the carpal end. Dr. Hutchinson, of Utica, saw this patient with me at the time, and diagnosed the injury as stated.

The fracture was reduced, and held in position by compress, secured by adhesive straps. The patient used the hand in knitting on the 16th day after the injury, and the dressings were removed on the 18th day. A perfect cure resulted.

Frank Gauber, aged 58 years, fell from a tree, July 26, 1872, producing a fracture of the inferior Maxillary bone and a fracture of the right radius ¾ of an inch above the wrist joint, with dislocation of the carpal end of the ulna. This patient also had a concussion of the dorsal vertebra, with other severe injuries.

The injury to the fore-arm was reduced by manipulations, after the plan of Dr. Moore, and secured by compress and adhesive plaster bandage, in the usual plan of his new method of treating such injuries. On the thirty-fifth day the dressings were removed, and the cure perfect.

In this case the dressings were left on longer than required, except as a caution, on account of the restless state of the patient.

Mrs. A. P. F., of Troy, N. Y., aged about 50 years, fell from a chair while standing on it, Sept. 3d, 1872, at the Clinton Hotel in this City, striking on the palm of the left hand, fracturing the radius one inch from the carpal end of the bone and dislocating the ulna at the lower end.

This case was treated as the preceding one, and the patient was sent to her home in Troy on the third day after the injury. The subsequent treatment of the case was rendered by my friend Dr. H. B. Whiton, of that city.

The result of the cure was perfect, as is shown by a letter I received from the Doctor, under date of Sept. 27. He says "your case of colles fracture has resulted in a perfect cure and this day I removed the dressings." You will observe that the cure was completed on the twenty-fourth day after the injury. The doctor further says in the letter refeired to, "I was on the business committee of the State Society when Dr. Moore read his paper, and am glad to have seen this and other cases confirming his simple and beautiful treatment; I think many who have not seen such perfect cures result from other modes of treatment, would be tempted to deny that there had been a fracture."

A lad aged 13 year, while at play with his fellows, fell from an eminence striking on his hands. He was immediately brought into my office, and the injury readily discovered. The right radius was fractured nearly one inch above the wrist. The evidence was slight crepitus, deformity and pain.

The treatment was reduction, secured by compress and adhesive plaster. This lad suffered but little from the injury, and on the fourteenth day the dressing was removed and the cure perfect.

Sept. 26th, 1872, Mrs. W., aged over 60 years, received a blow striking the left form-arm on the posterior side two inches above the wrist fracturing the radius at that point. Severe inflammation of the joint and hand followed the injury. Then very extensive swelling, redness and pain, which continued two weeks in an acute pain. The fracture after the reduction was treated after Dr. Moore's plan, and the union is perfect without deformity, though up to this time the patient has been unable to use the hand. The result of this case is due more to the inflammation than to the fracture. The inflammation of the hand was of a sub-acute form and presented a different appearance from that usually seen in those cases of colles fracture which terminate in deformity or an imperfect cure. The hand is puffy and tender, though there is perfect mobility of all the joints by manual force.

I am fully convinced by my own experience of the value of the simple and effective treatment presented by Prof. Moore.

